# Incidence and Prevalence of Major Central Nervous System Involvement in Systemic Lupus Erythematosus: A 3-Year Prospective Study of 370 Patients

**DOI:** 10.1371/journal.pone.0055843

**Published:** 2013-02-12

**Authors:** Eleni I. Kampylafka, Haralampos Alexopoulos, Michalis L. Kosmidis, Demosthenes B. Panagiotakos, Panayiotis G. Vlachoyiannopoulos, Marinos C. Dalakas, Haralampos M. Moutsopoulos, Athanasios G. Tzioufas

**Affiliations:** 1 Department of Pathophysiology, School of Medicine, National and Kapodistrian University of Athens, Athens, Greece; 2 Department of Nutrition and Dietetics, Harokopio University, Athens, Greece; Hospital Nacional de Parapléjicos - SESCAM, Spain

## Abstract

**Background:**

The incidence and prevalence of CNS involvement in SLE remains unclear owing to conflicting results in the published studies. The aim of the study was to evaluate the incidence and prevalence of major definite CNS events in SLE patients.

**Methods:**

370 SLE patients with no previous history of CNS involvement were prospectively evaluated in a tertiary hospital referral center for 3 years. Major CNS manifestations were codified according to ACR definitions, including chorea, aseptic meningitis, psychosis, seizures, myelopathy, demyelinating syndrome, acute confusional state and strokes. Minor CNS events were excluded. ECLAM and SLEDAI-SELENA Modification scores were used to evaluate disease activity and SLICC/ACR Damage Index was used to assess accumulated damage.

**Results:**

16/370 (4.3%) patients presented with a total of 23 major CNS events. These included seizures (35%), strokes (26%), myelopathy (22%), optic neuritis (8.7%), aseptic meningitis (4.3%) and acute psychosis (4.3%). Incidence was 7.8/100 person years. Among hospitalizations for SLE, 13% were due to CNS manifestations. Epileptic seizures were associated with high disease activity, while myelopathy correlated with lower disease activity and NMO-IgG antibodies *(P≤0.05)*. Stroke incidence correlated with APS coexistence *(P = 0.06)*. Overall, CNS involvement correlated with high ECLAM and SLEDAI scores *(P<0.001)*.

**Conclusions:**

Clinically severe CNS involvement is rare in SLE patients, accounting for 7.8/100 person years. CNS involvement correlates with high disease activity and coexistence of specific features that define the respective CNS syndromes.

## Introduction

Systemic Lupus Erythematosus (SLE) is an autoimmune systemic disease which has a prevalence of 52.2/100.000 in the United States [1]. Neurological involvement in the morbidity and mortality of SLE is considered significant [2]–[4] and many studies have tried to determine the incidence and prevalence of neurologic manifestations. Major obstacles have been the often non-specific nature of these manifestations and the scarcity of specific laboratory tests. In addition, the need to differentiate Central Nervous System (CNS) involvement attributed to SLE from existing co-morbidities, drug adverse reactions or infections [5], [6] had generated further diagnostic dilemmas [7]. In 1999, the American College of Rheumatology (ACR) formulated a nomenclature system which provided case definitions for 19 neuropsychiatric SLE syndromes[8].

Despite these advances, the reported frequency of neurologic involvement in SLE continues to vary greatly, ranging from 12% to 95% [9]–[12]. This discrepancy can be attributed to factors such as study design, diagnostic approach and patients’ baseline characteristics [10], [12]. Still, the main reason has been the inclusion of non-specific, minor symptoms including headaches, mild cognitive dysfunction and depression, which may occur in several chronic autoimmune diseases, treated with steroids and immunosuppressants [3], [13], [14].

The aim of the present study was to evaluate the prevalence, annual incidence and the clinical factors associated with definite organic CNS manifestations, in a cohort of SLE patients.

## Materials and Methods

### Study’s Sample

The study was conducted between July 1^st^ 2008 and July 31^st^ 2011 in the Department of Pathophysiology, University of Athens. 370 SLE patients (32±14 years, 88% females) with no history of CNS involvement attributed to SLE were consecutively included. All patients fulfilled the 1997 ACR classification criteria for SLE [15] and diagnosis was upon recognition of ≥4 ACR criteria.

Patients were informed about the scope and procedures of the study and provided written informed consent. The study was approved by the Scientific Council of “Laikon” University Hospital and by the Ethical Committee of the Medical School.

### Definition of the Organic CNS Involvement

Among the 19 ACR case definitions [8], we selected 8 objective major CNS manifestations. These were chorea, aseptic meningitis, psychosis, seizures, myelopathy, demyelinating syndrome, acute confusional state and strokes. Diagnosis was based on neurological examination, laboratory and imaging studies, and the exclusions and associations proposed in the ACR glossary [8]. CNS manifestations occurring in patients with recent cancer diagnosis (<5 years), non-controlled hypo- or hyperthyroidism, active CNS or systemic infection, HBV or HCV infection, and in patients who had received medications with potential neurological adverse reactions prior to the onset of the CNS manifestations, were excluded.

All cases of Peripheral Nervous System (PNS) involvement were excluded. Anxiety, headaches, subclinical cognitive dysfunction and mild depression were also excluded, as they lack SLE specificity [3], [13], [16]. Subclinical cognitive dysfunction is not an uncommon finding among SLE patients with or without overt CNS manifestations [17], [18] and also appears in the Antiphospholipid Syndrome (APS) [19]. Searching for non self-reported cognitive deficits was beyond the scope of our study. Therefore, no detailed neuropsychological battery was applied. The Mini Mental Scale Examination (MMSE) [20], [21] was performed in all patients with self-reported cognitive deficits during neurological examination, and overt cognitive dysfunction was defined as a MMSE score of <25.

### Clinical and Laboratory Assessment

All major CNS manifestations attributed to SLE that occurred during the study period were recorded. Upon first visit, patients gave a medical history and underwent clinical and neurological examination, including routine laboratory and immunological testing. Demographic data were recorded. European Consensus Lupus Activity Measurement score (ECLAM) [22] and Systemic Lupus Erythematosus Disease Activity Index (SLEDAI) - SELENA Modification [23], were used to evaluate disease activity. The SLICC/ACR Damage Index (SDI) [24] was used to assess accumulated damage due to the disease. As ECLAM, SLEDAI and SDI scores include neurological variables they were also calculated after removing these variables. The indexes without neurological variables are referred to as modified ECLAM, SLEDAI and SDI (m-ECLAM, m-SLEDAI and m-SDI, respectively). The types of glomerulonephritis were classified according to the 2003 International Society of Nephrology/Renal Pathology Society (ISN/RPS) classification [25]. Secondary APS was diagnosed according to the 2006 update of the 1999 ACR criteria [26]. Patients were evaluated every 4 to 8 months or every time a disease relapse or complication occurred. Hospitalizations, irrespective of the overriding symptom, were recorded. A clinical and neurological examination by the same expert neurologist and CSF examinations/Magnetic Resonance Imaging (MRI)/Electroencephalogram (EEG) were conducted whenever a CNS event occurred. The presence of Antinuclear Antibodies (ANA) and anti-Ribosomal P antibodies [27] was detected by Indirect Immunofluorescence (IIF) on Hep-2 cells, while NMO-IgG/anti-AQP4 antibodies were detected by IIF on mouse brain tissue and a cell based assay [28], [29]. ECLAM, SLEDAI and SDI scores were calculated at the occurrence of every new CNS event.

### Statistical Analysis

Continuous variables are presented as mean ± SD when normally distributed, or as median and quartiles when skewed. Categorical variables are presented as frequencies. Normality was graphically tested using P-P plots. The chi-square test (categorical variables) and the Mann-Whitney test (quantitative variables) were used to evaluate associations between subgroups of patients with CNS involvement. Incidence was evaluated among newly diagnosed SLE patients using the person years of observation. Binary logistic regression models were applied to evaluate associations of patients’ characteristics with CNS involvement. Results are presented as odds ratios and the corresponding 95% confidence intervals. Hosmer-Lemeshow statistics were used to evaluate all models’ goodness of fit. The level of statistical significance was *P≤0.05*. SPSS statistical software was used for analyses (SPSS Hellas Inc., Athens, Greece).

## Results

### Prevalence and Incidence

A total of 370 SLE patients with no prior CNS manifestations were enrolled. Patients were predominantly female (88%), with a mean age of 32±14 and mean disease duration of 9±7.8 years. Demographic and clinical characteristics are presented in *Table 1*. After excluding the non-specific minor CNS complaints (n = 76 patients), the prevalence of major CNS events among SLE patients was 4.3%; 16/370 patients presented a total of 23 CNS events. The 23 CNS events were: seizures (n = 8, 35%), strokes (n = 6, 26%), myelopathy (n = 5, 22%), optic neuritis (n = 2, 8.7%), aseptic meningitis (n = 1, 4.3%) and acute psychosis (n = 1, 4.3%). All seizures were generalized tonic–clonic, while 3 patients evolved to *status epilepticus*. All strokes were ischemic. Two patients had 2 or more, small ischemic lesions, 2 patients had a large infarct due to thrombosis of a central artery, while one patient manifested multiple transient ischemic attacks in the course of days. Optic neuritis presented as part of Neuromyelitis Optica. Chorea, acute demyelinating disease and acute confusional state were not observed during the 3-year observation period. Finally, no patients were defined as having overt cognitive deficits (MMSE <25).

**Table 1:** Demographic and clinical characteristics of the SLE patients.

Patient Characteristics	All SLE patients (n = 370)	Patients with CNS Involvement (n = 16)
Age at diagnosis *(m ± SD)*	32±14	30±12
Sex		
Male, *n (%)*	43 (12%)	1 (6.3%)
Female, *n (%)*	327 (88%)	15 (94%)
Disease duration in years (*m ± SD)*	9±7.8	5.7±8.2
ECLAM score *(m ± SD)* ^†^	1.6±1.8	4.6±3
ECLAM score at end of follow up *(m ± SD)*	*NA*	0.4±0.7
SLEDAI score *(m ± SD)* ^†^	3.6±5.5	18.1±9.9
SLEDAI score at end of follow up *(m ± SD)*	*NA*	0.9±1.4
SDI score at CNS Onset *(m ± SD)*	*NA*	1.4±0.8
SDI score at end of follow up *(m ± SD)*	*NA*	2.1±1.2
Secondary APS, *n (%)*	47 (13%)	4 (25%)
Individual Disease Manifestations**		
Serositis, *n (%)*	96 (26%)	6 (38%)
Pleurisy, *n (%)*	41 (11%)	5 (31%)
Pericarditis, *n (%)*	40 (11%)	5 (31%)
Arthritis, *n (%)*	174 (47%)	16 (100%)
Glomerulonephritis, *n (%)*	164 (44%)	7 (44%)
Class I, *n (%)*	5 (1.4%)	0 (0%)
Class II, *n (%)*	42 (11%)	2 (13%)
Class III, *n (%)*	63 (17%)	2 (13%)
Class IV, *n (%)*	37 (10%)	1 (6.3%)
Class V, *n (%)*	42 (11%)	3 (19%)
Class VI, *n (%)*	4 (1.1%)	0 (0%)
CNS involvement, *n (%)*	16 (4.3%)	16 (100%)
Hematologic disorder, *n (%)*	368 (66%)	10 (63%)
Serological Manifestations**		
ANA, *n (%)*	361(96%)	15 (94%)
Anti-dsDNA antibodies, *n (%)*	345 (70%)	11 (69%)
acL antibodies, *n (%)*	314 (40%)	8 (50%)
Anti-Rib P antibodies, *n (%)*	*Not determined*	2 (13%)
NMO-IgG antibodies, *n (%)*	*Not determined*	2 (13%)
Neurological Manifestations**		
Epileptic Seizures, *n (%)*	6 (1.6%)	6 (38%)
Strokes, *n (%)*	5 (1.4%)	5 (31%)
Myelopathy, *n (%)*	4 (1.1%)	4 (25%)
Optic Neuritis, *n (%)*	1 (0.3%)	1 (6.3%)
Psychosis, *n (%)*	1 (0.3%)	1 (6.3%)
Aseptic Meningitis, *n (%)*	1 (0.3%)	1 (6.3%)
1^st^ CNS episode at SLE diagnosis, *n (%)*	*NA*	7 (44%)
Patients with relapsing CNS episodes, *n (%)*	*NA*	4 (25%)
Patients with >1 simultaneous CNS events, *n (%)*	*NA*	2 (13%)
Disease Manifestations Concurrent to CNS Onset		
Serositis, *n (%)*	*NA*	3 (19%)
Arthritis, *n (%)*	*NA*	16 (100%)
Glomerulonephritis, *n (%)*	*NA*	5 (31%)
Hematologic disorder, *n (%)*	*NA*	10 (63%)
Medications**		
Corticosteroids, *n (%)*	339 (92%)	16 (100%)
Azathioprine, *n (%)*	131 (35%)	4 (25%)
Hydroxychloroquine, *n (%)*	231 (62%)	7 (44%)
Methotrexate, *n (%)*	64 (17%)	2 (13%)
Cyclophosphamide, *n (%)*	123 (33%)	11 (69%)
Mycophenolate Mofetil, *n (%)*	119 (32%)	8 (50%)
Rituximab, *n (%)*	35 (9.5%)	5 (31%)

Seven out of 16 patients had CNS involvement upon diagnosis (44%), while 4 patients (25%) had a relapse of their initial CNS manifestations within the 3-year period. Two out of 16 patients (13%) had concurrent CNS manifestations; one presented with myelitis and optic neuritis, and the other with aseptic meningitis and psychosis. No death occurred in patients with CNS involvement. The all-cause mortality rate of the entire cohort (*n* = 370) during the follow up period was 0.5%. Demographic and clinical characteristics of SLE patients with CNS involvement are presented in *Table 1*.

Finally, the annual incidence of major CNS events among newly diagnosed SLE patients was 7.8 events/100 person-years (i.e., 8 cases out of 55 SLE patients).

### Hospitalization Rates

Eighty-seven out of the 370 SLE patients were admitted to the hospital 141 times during the 3-year follow-up period. 29 patients were hospitalized more than once. The most common hospitalization cause was a lupus flare (59%), while the second more frequent cause was an infection (20%). No CNS infections occurred. Flares included glomerulonephritis (27%), cytopenias (15%), CNS involvement (13%), musculoskeletal symptoms (8.5%), serositis (5.7%), hepatosplenomegaly and lymph node enlargement (4.3%), pneumonitis (3.5%), pancreatitis, colitis and myocarditis (<1%). Nearly one third (33%) of disease flares presented with more than one overlapping SLE manifestations. Overall, 16 SLE patients were admitted 18 times for neurological manifestations *(* *Table 2* *)*.

**Table 2:** Hospitalization causes of SLE patients during the 3 year follow up period.

Cause of Hospitalization	Hospitalizations (n)	Percentage (%)
Relapse**	83	59%
Glomerulonephritis	38	27%
Hematological	21	15%
CNS	18	13%
Musculosceletal	12	8.5%
Serositis	8	5.7%
Hepatosplenomegaly & Lymph Node Enlargement	6	4.3%
Pneumonitis	5	3.5%
Pancreatitis	3	<1%
Colitis	3	<1%
Myocarditis	1	<1%
Infection	28	20%
Other	30	21%
*(Chronic Renal Insufficiency, Cancer, Budd-Chiari Syndrome, alveolar hemorrhage, pulmonary hypertension)*		*(All less than 1%)*
**Total**	**141**	**100%**

** Relapse manifestations can coexist during the same hospitalization.

### Clinical Associations

We evaluated clinical and serological associations with specific neurological manifestations for the 16 SLE patients with CNS involvement. Epileptic seizures were associated with high ECLAM disease activity score *(P = 0.02)*, late onset during the disease course *(P = 0.03)*, previous history of serositis *(P<0.01),* and glomerulonephritis *(P = 0.03)*. Glomerulonephritis also tended to occur concurrently to the CNS events *(P = 0.03)*, though no correlations with any specific types of glomerulonephritis were found (*all P-values >0.10*). Further, two clinical associations were of interest; epileptic seizures tended to associate with high SLEDAI score *(P = 0.07)* and younger age at disease onset *(P = 0.08)*.

**Figure 1:**
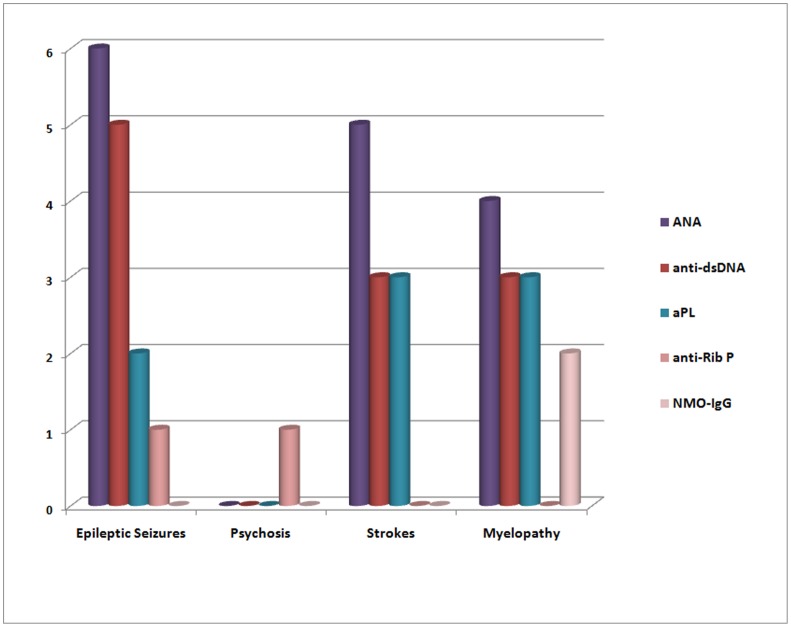
Numbers of patients with the respective antibodies within each CNS subgroup. Each column group refers to the respective CNS subgroup. Each column represents a discrete autoantibody, while Y axes displays the number of patients presenting with the respective autoantibody.

Myelopathy correlated with lower disease activity scores, low complement and NMO-IgG antibodies *(all P-values <0.05)*. Two patients with myelitis (2/4, 50%) were NMO-IgG/anti-AQP4 positive; the first had Neuromyelitis Optica (NMO) [30], while the other had Longitudinal Extensive Transverse Myelitis (LETM), which belongs to the NMO spectrum of disorders [31]. Presence of strokes tended to associate with secondary APS *(P = 0.06)* and was associated with higher SDI scores *(P = 0.03)*. One patient with acute psychosis was positive only for anti-ribosomal P antibodies (*Figure 1*).

Analysis was repeated after removing neurological variables from ECLAM, SLEDAI and SDI scores. All associations previously observed with ECLAM remained significant *(P<0.05)* after modification (m-ECLAM). The m-SLEDAI score tended to inversely correlate with myelitis *(P = 0.08)* but did not correlate with epileptic seizures *(P = 0.23)*. m-SDI did not correlate with strokes *(P = 0.21)*.

The 16 patients with CNS involvement were compared to the remaining 354 SLE patients without neurologic manifestations. The results from univariate analyses calculating the association of demographic and clinical characteristics with CNS involvement are presented in *Table 3*. Since residual confounding may exist, multiple logistic regression analysis was used to calculate the association of ECLAM and SLEDAI scores, modified ECLAM and SLEDAI scores, aCL antibodies, secondary APS, arthritis, serositis and glomerulonephritis coexistence with CNS involvement, after adjusting for age, sex, and disease duration. ECLAM and SLEDAI scores were mutually replaced in the model due to high inter-correlation. The same mutual replacement was conducted regarding m-ECLAM and m-SLEDAI, as well as APS coexistence and aCL antibodies. Arthritis and glomerulonephritis were excluded from the SLEDAI models, due to high inter-correlation. Both unmodified and modified ECLAM and SLEDAI scores were positively associated with increased likelihood of CNS involvement *(Odds ratio per 1 unit increase in* ECLAM = *2.077, 95%CI 1.55–2.79, Odds ratio per 1 unit increase in* SLEDAI = *1.39, 95%CI 1.24–1.56, Odds ratio per 1 unit increase in m-*ECLAM = *1.56, 95%CI 1.19–2.05, Odds ratio per 1 unit increase in m-*SLEDAI = *1.26, 95%CI 1.16–1.41),* after adjusting for APS coexistence and the other factors mentioned above. Similar results were revealed when aCL antibodies replaced APS coexistence in the previous models (data not shown). Moreover, in the models that SLEDAI score was included, APS coexistence was associated with higher likelihood of CNS involvement *(Odds ratio per 1 unit increase in* SLEDAI = *0.16, 95%CI 0.03–0.87, Odds ratio per 1 unit increase in m-*SLEDAI = *0.26, 95%CI 0.07–1.04)*, a finding that was not confirmed in models that included the ECLAM score instead of SLEDAI.

**Table 3:** Demographic and clinical characteristics of *n* = 370 SLE patients by CNS involvement group.

Variables	SLE patients with CNS Involvement (*n* = 16)	SLE patients without CNS Involvement (*n* = 354)	*P*
Age at Disease Onset	30±12	32±14	*0.48*
Sex			*0.70*
Male	1 (6.3%)	42 (12%)	
Female	15 (94%)	312 (88%)	
Disease Duration (yrs)	5.7±8.2	9.2±7.8	*0.009*
aCL antibodies	8 (50%)	140 (40%)	*0.60*
Secondary APS‡	4 (25%)	43 (12%)	*0.13*
ECLAM score	4.6±3	1.4±1.5	*<0.01*
m-ECLAM score†	3.3±2.5	1.4±1.5	*0.01*
SLEDAI score	18±9.9	3.1±4.1	*<0.01*
m-SLEDAI score†	12±8.1	3.1±4.1	*<0.01*
Arthritis	16 (100%)	170 (48%)	*0.07*
Glomerulonephritis	7 (44%)	157 (44%)	*1.00*
Serositis	6 (38%)	90 (25%)	*0.38*

*P*-values derived through chi-square test for the comparisons of sex, existence of aCL antibodies, secondary APS, arthritis, glomerulonephritis and serositis, and through Mann-Whitney U-test for the comparisons of age at disease onset, disease duration, ECLAM, SLEDAI, m-ECLAM and m- SLEDAI scores.

† m-ECLAM and m-SLEDAI are the modified scores after exclusion of the neurological variables

‡ APS, Antiphospholipid Syndrome.

## Discussion

This work was focused on the distinction of major CNS manifestations in SLE and studied their prevalence and incidence in a cohort of unselected SLE patients. It was shown, that severe CNS involvement is rather uncommon with a prevalence of 4.3% and an incidence rate of 7.8/100 person years. Moreover, ECLAM and SLEDAI scores and APS coexistence were positively associated with increased likelihood of CNS involvement. Despite the limitations of the present work, mainly due to the relatively small sample size, the aforementioned findings deserve further attention since they highlight the importance and rarity of major CNS events that can occur in the context of SLE.

CNS involvement can influence both damage accrual and mortality in SLE [32], [33]. This is mostly attributed to the major CNS manifestations of the disease, including seizures, myelopathy and strokes [2], [32], [34]. Furthermore, neurological involvement appears to be an independent risk factor for hospitalizations in SLE [35]. Our study reinforces these conclusions, as 13% (18/141) of SLE hospitalizations in our cohort were due to major CNS manifestations. An additional clinical observation is that 2 out of these 141 hospitalizations were in the Intensive Care Unit (ICU). Both were due to CNS involvement. The aforementioned findings underline the necessity for rapid diagnosis and treatment in patients presenting with major CNS manifestations.

The true incidence and prevalence of severe CNS involvement in SLE is often overshadowed by the inclusion of minor non-specific symptoms with no direct pathogenetic correlation with SLE, such as mild cognitive dysfunction, headache, mild depression, anxiety and paresthesias with negative electroneuromyography [9], [13]. In the present prospective study, PNS symptoms and all the aforementioned minor events [9], were excluded and stringent criteria to include only CNS manifestations directly attributed to SLE were applied. Moreover, in a 2004 meta-analysis it was shown that prevalence of all types of headaches in SLE patents was not different from that of healthy controls [36], while another study has shown that SLE patients without neurological manifestations exhibit a decline in attention, memory, and reasoning [17].

Eight major CNS syndromes namely epileptic seizures, strokes, myelopathy, psychosis, aseptic meningitis, acute confusional state, chorea and demyelinating syndrome, were included. Although optic neuritis is categorized by the ACR [8] within the PNS syndromes, in our study it was included as a CNS event since it occurred in the context of Neuromyelitis Optica.

The estimated prevalence of definite CNS involvement in SLE in our cohort was 4.3%, much lower compared to previous prospective studies in which minor events and PNS symptoms were included, where the prevalence ranged from 13% to 46% [4], [9], [16].

Unlike prevalence, incidence has not been previously calculated, although a recent review proposed a cumulative incidence of approximately 30–40% [10]. In our study, incidence was calculated among newly diagnosed SLE patients (n = 55) during the 3-year follow-up period, and was 7.8/100 person years. In published series, most neurologic events tend to accumulate early during the disease[4], [7], [10]. CNS involvement was also an early event in our cohort, as 44% of our patients manifested their CNS events simultaneously with disease onset.

Roughly 13% of hospitalizations during the 3-year period were due to SLE-related CNS manifestations, making them the third more common lupus associated cause for patient admission. Evidently, even though rare, severe CNS manifestations account for a notable percentage of hospital admissions in SLE patients.

A statistically significant correlation was found between high disease activity and CNS manifestations *(P<0.001)*, in line with other studies [10], [37], [38]. This conclusion was reinforced after evaluating our SLE patients using two different disease activity scores (ECLAM and SLEDAI) and repeating the analysis with the exclusion of neurological factors from the scores. Moreover, a statistically significant correlation was observed between secondary APS and CNS involvement *(P = 0.03)*. Other studies have also proposed a correlation between neurological manifestations in SLE and secondary APS [37], [39], [40].

In our cohort, two discrete clinical phenotypes were identified. The first was the diffuse phenotype that included epileptic seizures and psychosis. The second was focal and included myelopathy and strokes. The correlation of discrete neurological syndromes with other clinical and serological features of the disease revealed that epileptic seizures appeared in patients with higher disease activity scores (*P = 0.02* and *P = 0.07,* for ECLAM and SLEDAI scores respectively), when compared to other neurological manifestations. Furthermore, patients with epileptic seizures were younger at disease onset *(P = 0.08)*, in agreement with previous studies [41], and expressed the seizures later during the disease *(P = 0.03)*, unlike other studies where seizures appeared near the onset of the disease [42]. Glomerulonephritis was present more often in these patients and occurred concurrently to the onset of epileptic seizures *(P = 0.03)*. This observation is in line with a previous study, where epileptic seizures were a frequent manifestation in patients with lupus nephritis [43].

Three out of 6 patients who manifested epileptic seizures had characteristic features in their MRIs; two had Posterior Reversible Encephalopathy Syndrome (PRES) [44] and 1 had vasculitic-like lesions. All 3 patients had common clinical features; very high disease activity, active glomerulonephritis presenting as nephrotic syndrome, and symptom amelioration following immunosuppression and antiepileptic treatment. According to previous reports, two types of PRES in SLE are thought to exist; “hypertensive PRES,” being reversible with antihypertensive and anticonvulsive treatment concomitant with inactive SLE, and PRES requiring immunosuppressive therapy that could be considered a neurological manifestation of active SLE [44]. Finally, as seizures preceded the onset of the nephrotic syndrome, it is unlikely that they were due to nephrotic encephalopathy.

Myelopathy correlated with lower disease activity scores and the presence of NMO-IgG/anti-AQP4 antibodies (*P*≤0.05). Patients with both myelopathy and NMO-IgG antibodies (2/4) had NMO spectrum symptoms [31]. The first had Neuromyelitis Optica and the second had LETM. These entities have been previously found in the context of systemic diseases [45]. A recent study [46] described two discrete subtypes of lupus myelitis. The first appeared with gray matter dysfunction, irreversible paraplegia, higher SLE activity and a CSF profile resembling that of bacterial meningitis. The second subtype involved patients with white matter dysfunction and lower SLE activity, who were more likely to meet criteria for NMO. In our study, patients fell into the second myelitis subtype.

Strokes appeared more frequently in patients with secondary APS *(P = 0.06)* and a worse SDI *(P = 0.03)*. m-SDI did not correlate to strokes, leading to the assumption that strokes themselves could aggravate disease damage.

CNS involvement in SLE is due to multiple mechanisms, including microvasculopathy, autoantibody-mediated injury or intrathecal production of cytokines that enhance the blood-brain barrier permeability, thus allowing leukocytes and antibodies to enter the CNS [10]. Autoantibodies with strong clinical correlation to neurological disease in SLE are the aPL (observed in our study in 8/16 patients), the anti-ribosomal P antibodies (2/16 patients), and the NMO-IgG/anti-AQP4 antibodies that appeared in 2 out of 4 patients with myelopathy [10], [31], [47].

### Conclusions

Our study, despite certain limitations (single center, relatively short follow-up), focused on severe CNS involvement, attributed with certainty to SLE. Clinically severe CNS involvement appears to be relatively rare in SLE patients, correlates with disease activity and manifests with specific features, according to the respective CNS syndromes. Our work also highlights the necessity for speedy recognition and intensive treatment of such major events, due to their significant impact on SLE damage accrual and mortality. Multicenter and long-term follow up studies could be helpful to strengthen our conclusions.
